# Introducing Genes With Significant Role in Migraine: An Interactomic Approach

**DOI:** 10.32598/bcn.10.4.363

**Published:** 2019-07-01

**Authors:** Mona Zamanian Azodi, Mostafa Rezaei Tavirani, Reza Mahmoud Robati

**Affiliations:** 1. Student Research Committee, Shahid Beheshti University of Medical Sciences, Tehran, Iran.; 2. Proteomics Research Center, Faculty of Paramedical Sciences, Shahid Beheshti University of Medical Sciences, Tehran, Iran.; 3. Skin Research Center, Shahid Beheshti University of Medical Sciences, Tehran, Iran.

**Keywords:** Migraine disorders, Protein interaction maps, Genes

## Abstract

**Introduction:**

Migraine is a severe kind of headache with the chance hereditary of 50%. Molecular studies can promote understanding of migraine pathophysiology. One of which is bioinformatics approach that could provide additional information related to the identified biomarkers.

**Methods:**

In this research, migraine genes are studies in terms of interaction pattern to introduce important individuals. Through STRING database Plug-in in Cytoscape, candidate genes for migraine were retrieved and analyzed by related applications. Based on centrality and action types (expression, activation, and inhibition) genes were screened.

**Results:**

Numbers of 33 central genes including seven hub-bottlenecks were identified which 70% of central genes were involved in expression action with each other. Activation was dominate action relative to inhibition between the central genes.

**Conclusion:**

The finding indicates that insulin is the most important gene relative to migraine. It seems regulation of metabolism play critical role in control of migraine.

## Highlights

- Gene activation is the dominant actor in the interactome of migraine.
- Insulin has been found as the most significant related gene in migraine.
- Migraine could be a metabolism-dependent disorder.

## Plain Language Summary

Migraine pain disturbs the patient’s lifestyle. There is a lot of information about migraine, however, its management still needs much research. In this study, the molecular aspects of migraine are evaluated to find its controllable biological factors. Targeting these factors may be useful in migraine treatment. The findings of this study showed that metabolism control, especially insulin regulation, plays a critical role in migraine.

## 1. Introduction

Migraine is a complex episodic headache and is known as the sixth cause of reducing the quality of life condition according to the WHO report. Also, its incidence is higher in women (Goadsby et al., 2017). This type of headache is usually associated with some other problems, like nausea, vomiting, sensitivity to light, and sound that could lead to aura (Pellacani et al., 2016). High-frequency headache, medication overuse, obesity and several risk factors are important in migraine progression (Moriarty & Mallick-Searle, 2016). Understanding the mechanisms of this condition is still under investigation. The diagnosis of migraine is mostly based on clinical assessments (Shin, Shin, & Kim, 2017).

Pathophysiological studies of migraine suggest that it is associated with vascular system inflammation (Mastia et al., 2016). Molecular studies have identified some molecular agents in this regard such as different neuropeptides and cytokines such as C-Reactive Protein (CRP), Interleukin-1 (IL-1), IL-6, and Tumor Necrosis Factor-α (TNF-α) (Mastia et al., 2016). High throughput studies could be beneficial to introduce a large number of potential biomarkers in different kinds of diseases. There are some genomic, proteomic, and metabolomic analyses related to deciphering the risk of migraine. These approaches introduce potential biomarkers for migraine that can be useful for diagnosis and treatments (Gerring, Powell, Montgomery, & Nyholt, 2018; Mastithe et al., 2016; Shin et al., 2017; Tafuri et al., 2015).

Bioinformatics can also offer more in this regard. By analyzing genes in a whole interacting pattern, the role of each one could be better characterized. In fact, any changes in the phenotype is related to alteration in interaction of these molecules and in this way, a specific biological answer could be found. This biological response could cause abnormal behavior, which can be considered as a disease state (Tavirani et al., 2018).

In light of this phenomenon and via network analysis, many genes related to a specific disorder could be screened and analyzed to recognize critical genes which can be nominated as diagnostic agents or drug targets (Rezaei-Tavirani, Tavirani, & Rostami, 2018). In this study, genes corresponding to migraine are interacted, analyzed, and screened to find vital ones to address possible therapeutic biomarkers for this brain disorder.

## 2. Methods

### 2.1. Protein-protein interaction network analysis

The associated genes to migraine were obtained via disease query of STRING database and were interacted as Protein-Protein Interaction (PPI) network by Cytoscape version 3.6.0. The genes were connected by default condition via undirected edges. The main connected components of the PPI network were analyzed and visualized by the network analyzer plugin of Cytoscape.

The network was layout by degree values. The high-value degree nodes (degrees above Mean+SD) were recognized as hub-nodes. The nodes characterized by the high value of betweenness centrality (the top 5% of nodes) were highlighted as bottlenecks. The common nodes between hubs and bottlenecks were introduced as hub-bottlenecks. The central nodes, including hubs, bottlenecks, and hub-bottlenecks, were organized as a sub-network and analyzed to determine node properties.

### 2.2 Action network analysis

The action maps of the 33 central nodes were evaluated via CluePedia v1.5.0. The expression, activation, and inhibition relationships between the nodes were investigated to find the significant role of a node in controlling the other central nodes. The connections were considered as directional edges.

## 3. Results

A total of 600 genes associated with migraine were requested from disease query of STRING, but only 451 genes were found. Of them, 383 genes were included in PPI, and the other 68 genes were isolated.

**Figure 1.**
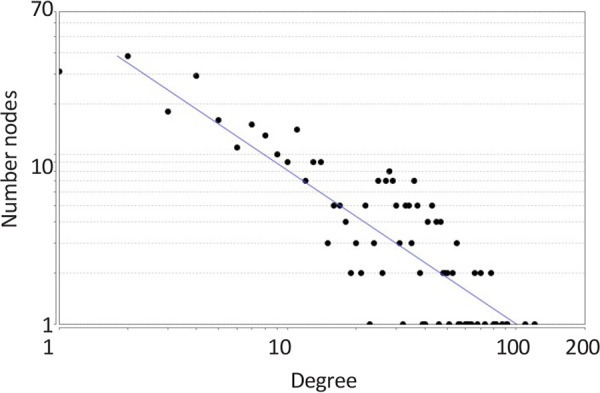
Degree distribution of migraine PPI network

**Figure 2.**
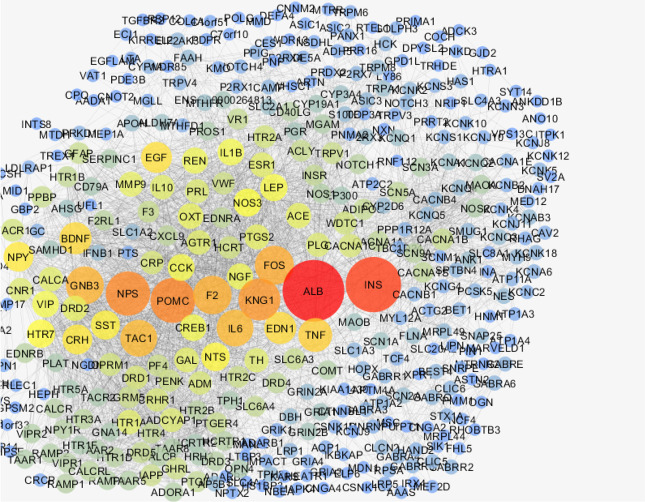
PPI network of migraine, nodes are layout by degree values

The network was analyzed, and degree of distribution (Figure 1) was fitted by y=axb were a, b, correlation, and R-squared were equal to 64.948, −0.904, 0.855, and 0.742, respectively. The results indicate a scale-free network. The network was visualized based on degree values (Figure 2). A total of 20 hubs and 20 bottlenecks were determined as well as 7 hub-bottlenecks (common between hubs and bottlenecks), so the total number was 33 genes (Table 1).

**Table 1.** The hubs (red), bottlenecks (green), and hub-bottlenecks (red-green) of migraine (BC refers to betweenness centrality).

**R**	**Name**	**Description**	**Degree**	**BC**
1	ALB	Albumin	121	0.131298
2	INS	Insulin	110	0.125716
3	POMC	Proopiomelanocortin	92	0.018258
4	NPS	Neuropeptide S	91	0.02901
5	KNG1	Kininogen 1	87	0.019268
6	FOS	FBJ murine osteosarcoma viral oncogene homolog	82	0.019946
7	F2	Coagulation factor II (thrombin)	81	0.018713
8	GNB3	Guanine nucleotide binding protein (G protein), beta polypeptide 3	80	0.042513
9	IL6	Interleukin 6 (interferon, beta 2)	78	0.020569
10	TAC1	Tachykinin, precursor 1	78	0.016064
11	TNF	Tumor necrosis factor	73	0.020693
12	BDNF	Brain-derived neurotrophic factor	70	0.024804
13	EDN1	Endothelin 1	70	0.018413
14	EGF	Epidermal growth factor	68	0.021209
15	CRH	Corticotropin-releasing hormone	66	0.006211
16	NPY	Neuropeptide Y	66	0.006144
17	SST	Somatostatin	64	0.010855
18	NTS	Neurotensin	62	0.02408
19	HTR7	5-Hydroxytryptamine (serotonin) receptor 7, adenylate cyclase-coupled	61	0.011028
20	NOS3	Nitric oxide synthase 3 (endothelial cell)	60	0.019226
21	CREB1	cAMP responsive element binding protein 1	55	0.024585
22	MMP9	Matrix metallopeptidase 9 (Gelatinase B, 92kDa gelatinase, 92kDa type IV collagenase)	49	0.027458
23	TH	Tyrosine hydroxylase	46	0.028666
24	PRL	Prolactin	45	0.02854
25	CACNA1C	Calcium channel, voltage-dependent, L type, alpha 1C subunit	43	0.038877
26	WDTC1	WD and tetratricopeptide repeats 1	41	0.026239
27	ACLY	ATP citrate lyase	36	0.052981
28	CACNA1B	Calcium channel, voltage-dependent, N type, alpha 1B subunit	36	0.028122
29	CACNA1A	Calcium channel, voltage-dependent, P/Q type, alpha 1A subunit	33	0.027352
30	NOTCH1	Notch 1	30	0.023984
31	ALDH7A1	Aldehyde dehydrogenase 7 family, member A1	19	0.025062
32	CLCN2	Chloride channel, voltage-sensitive 2	13	0.027591
33	GABRQ	γ-Aminobutyric Acid (GABA) A receptor, theta	13	0.023171

**Figure 3.**
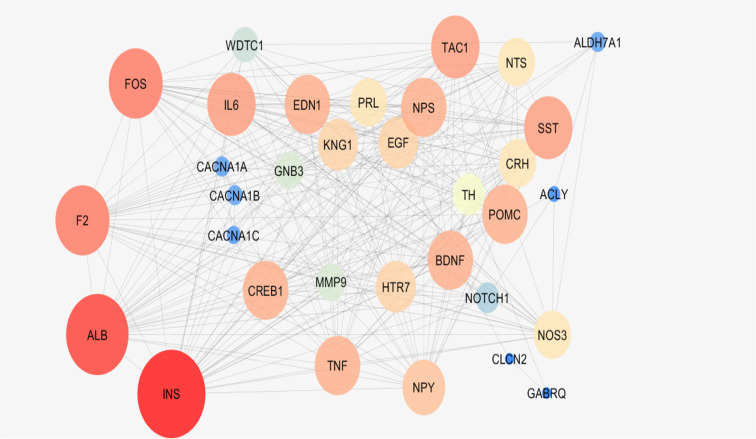
A sub-network of migraine PPI network.The nodes are layout based on degree value

**Figure 4.**
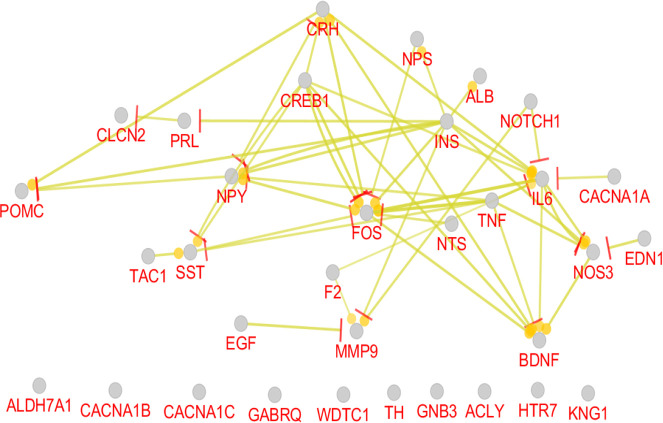
Expression pattern of 33 nodes of migraine PPI network. The round tips and the vertical bar tips refer to up-regulation and down-regulation, respectively.

**Figure 5.**
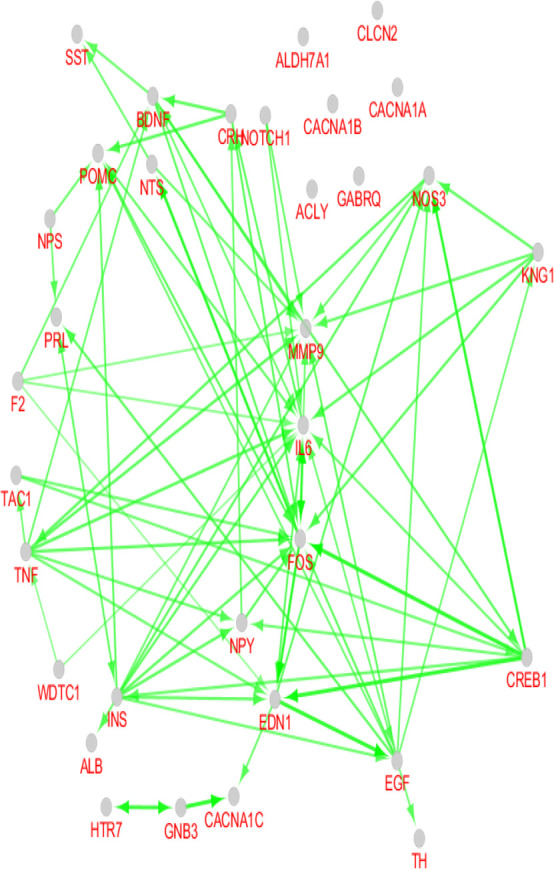
Activation action of 33 nodes of migraine PPI network arrow direction refers to activation direction

**Figure 6.**
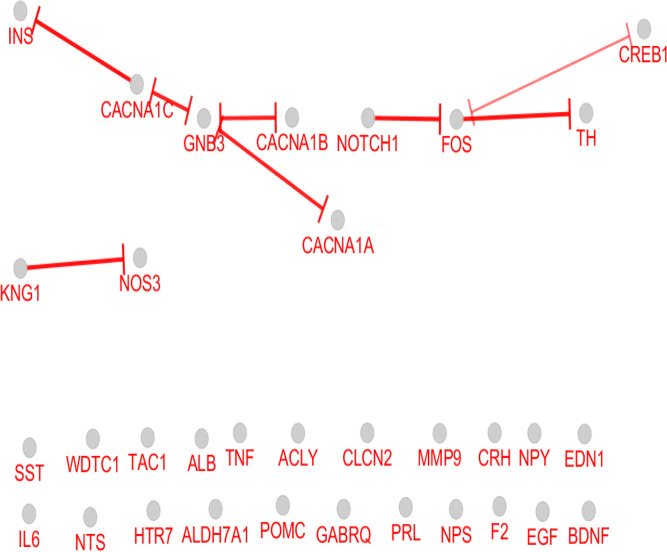
Inhibition relationships between 33 nodes of migraine PPI network vertical bar tips refer to inhibition direction

As it is depicted in Table 1, there are 7 hub-bottlenecks, including ALB, INS, NPS, GNB3, BDNF, EGF, and NTS. Since the hubs, bottlenecks, and hub-bottlenecks play different roles in the network, a sub-network, including 33 genes, was constructed (Figure 3). The central parameters, including the degree and betweenness of sub-network nodes, were determined and shown in Table 2. As it is shown in Figures 4, 5 and 6, the action patterns of expression, activation, and inhibition connections for 33 central nodes are presented. Directional edges connect nodes and refer to action mode. In some cases, the former node acts on the other connected node (nodes) while there are cases which a node affects the other nodes and in turn be affected by the concerned node.

**Table 2.** Centralities of nodes of sub-network.

**R**	**Name**	**Description**	**Degree**	**BC**	**R1**
1	INS	Insulin	29	0.128	2
2	ALB	Albumin	27	0.061	1
3	FOS	FBJ murine osteosarcoma viral oncogene homolog	24	0.016	6
4	F2	Coagulation factor II (thrombin)	24	0.018	7
5	TAC1	Tachykinin, precursor 1	22	0.010	10
6	IL6	Interleukin 6 (interferon, beta 2)	22	0.021	9
7	SST	Somatostatin	22	0.010	17
8	TNF	Tumor necrosis factor	21	0.010	11
9	NPS	Neuropeptide S	21	0.007	4
10	EDN1	Endothelin 1	21	0.022	13
11	BDNF	Brain-derived neurotrophic factor	21	0.020	12
12	POMC	Proopiomelanocortin	21	0.007	3
13	CREB1	cAMP responsive element binding protein 1	21	0.020	21
14	NPY	Neuropeptide Y	20	0.006	16
15	KNG1	Kininogen 1	19	0.007	5
16	HTR7	5-Hydroxytryptamine (serotonin) receptor 7, adenylate cyclase-coupled	19	0.010	19
17	EGF	Epidermal growth factor	19	0.008	14
18	PRL	Prolactin	18	0.062	24
19	NTS	Neurotensin	18	0.007	18
20	NOS3	Nitric oxide synthase 3 (endothelial cell)	18	0.020	20
21	CRH	Corticotropin releasing hormone	18	0.004	15
22	TH	Tyrosine hydroxylase	16	0.060	23
23	MMP9	Matrix metallopeptidase 9 (gelatinase B, 92kDa gelatinase, 92kDa type IV collagenase)	14	0.002	29
24	GNB3	Guanine nucleotide binding protein (G protein), beta polypeptide 3	14	0.019	8
25	WDTC1	WD and tetratricopeptide repeats 1	13	0.005	26
26	NOTCH1	Notch 1	11	0.005	30
27	CACNA1B	Calcium channel, voltage-dependent, N type, alpha 1B subunit	6	0.003	28
28	CACNA1A	Calcium channel, voltage-dependent, P/Q type, alpha 1A subunit	6	0.002	29
29	ALDH7A1	Aldehyde dehydrogenase 7 family, member A1	6	0.002	31
30	CACNA1C	Calcium channel, voltage-dependent, L type, alpha 1C subunit	5	0.001	25
31	ACLY	ATP citrate lyase	4	0.001	27
32	CLCN2	Chloride channel, voltage-sensitive 2	2	0.001	32
33	GABRQ	γ-Aminobutyric cid (GABA) A receptor, theta	2	0.001	33

BC refers to betweenness centrality, R1 indicates to row position in Table 1, the colored nodes are the hub-bottlenecks of migraine PPI network.

## 4. Discussion

Based on the findings, 451 genes are associated with migraine accessible in STRING database, indicating many investigations on the genetics of migraine. As it is shown in Figures 1 and 2, there is a scale-free network related to migraine. In scale-free networks, a few nodes can be considered as critical nodes. PPI network analysis is a suitable method to screen many genes and select the important ones. As it is shown in Table 1, there are 20, 20, and 7 hubs, bottlenecks, and hub-bottlenecks, respectively in the migraine network. Hubs and bottlenecks are major players in networks and consequently in diseases.

On the other hand, hub-bottlenecks are critical elements of the network, with high impact in onset, and development of diseases. It seems that the seven introduced hub-bottlenecks are the crucial genes that are involved in migraine. As it is presented in Table 1, the hub-bottlenecks account for 21% of total hubs and bottlenecks. It may be the number of potent hubs or bottlenecks that are ignored as crucial genes due to exclusion from hub-bottlenecks. Bottleneck nodes play a critical role in controlling the network (Rezaei-Tavirani, Rezaei-Tavirani, Ahmadi, Naderi, & Abdi, 2017). To avoid this disadvantage, these 33 critical genes interacted and centrality parameters of interacted nodes were analyzed (Figure 3 and Table 2). The elements of Table 2 were considered for more analysis; however, only 24 top genes, including hub-bottlenecks of Table 1, are selected. Expression, activation, and inhibition are three important actions of genes which indicate the impact of genes on the molecular mechanism of diseases.

As it is shown in Figure 4, 23 genes (70% of 33 central genes) are involved in expression action network. A total number of 6 hub-bottlenecks (85% of critical genes) are included in the network. Of 7 critical genes, only GNB3 was isolated and did not play a role in the network. The most important regulatory gene in this network is INS. It has 6 up-regulating and 4 down-regulating connections with the elements of the network. INS as a robust regulatory gene regulates 8 genes (two genes are up and down-regulated by INS).

The second important gene is CREB1, which controls the expression of 5 genes in the network. Surprisingly, ALB, which is the first and second node in Tables 1 and 2, has no regulatory effect in the expression action network. This gene is up-regulated by the only connection that is connected to INS. FOS, the other highly related gene, is linked to 10 nodes. As it is discussed, INS and CREB1 have merely regulatory connections, but FOS is regulated mostly by the other genes. TNF regulates 7 genes, while no gene regulates it.

As it is shown in Figures 5 and 6, the most contributing genes in the network are activator genes. Among 33 genes, 27 ones, including all critical genes are involved in activation action network while 11 genes (33% of central genes) construct inhibition action network and among them, only 9 genes are inhibitor genes. INS and GNB3, and 30% of critical genes play inhibition role in action network. Like the expression map, the activation and inhibition networks show no significant role for ALB. It seems that albumin as an important career in the body is related to many genes but have no significant gene regulatory effects, which matches with its role in body.

The most activation arrows target FOS, IL6, and MMP9. Complex relationships between nodes of activation action network versus inhibition network refer to increase in biochemical activities of migraine. Inhibition action network is constructed by three separate parts which are organized as KNG1-NOS3, calcium channel-1 subunits-GNB3-INS, and NOTCH1-FOS-TH-CREB1.

Several studies support insulin sensitivity alteration in migraine, that results in increasing glucose concentration (Cavestro et al., 2007; Rainero et al., 2005). MU Jang et al. reported a significant correlation between a high level of neuropeptides and pain in migraine patients (Jang, Park, Kho, Chung, & Chung, 2011). Based on the report of MTA Tanure et al. BDNF level of migraine patients increases significantly while the TNF level does not change (Tanure, Gomez, Hurtado, Teixeira, & Domingues, 2010).

It is reported that TNF level changes in migraine patients without aura while it is normal in patients with chronic type tension headache (Covelli et al., 1990). Nitric oxide can be considered as a pain stimulus in migraine due to its role in vasodilation, but investigation indicates no significant correlation between nitric oxide synthase change and migraine (Griffiths, Nyholt, Curtain, Goadsby, & Brimage, 1997). NOS3 appears as the last hub-gene and is not a hub-bottleneck in our analysis. NOS3, as the most regulated gene in three action networks, did not show significant regulatory effects and was inhibited by KNG1. Increased activity of matrix metalloproteinases in migraine patients is confirmed, which MMP9 action as an initiator for this cascade.

Investigations show that matrix metalloproteinases can kill neurons via interfering with TNF receptors or creating neurotoxic chemokines. It is possible that matrix metalloproteinase inhibitors affect IL6 receptors (Lakhan & Avramut, 2012). Activated transcription factor CREB can activate c-FOS (that is known as a neuronal activation marker) and positively regulate BDNF expression. Over-expression of BDNF is confirmed in migraine patients (Guo, Deng, Bo, & Yang, 2017; Tanure et al., 2010). As it is shown in Figure 5, activation actions of CREB1-FOS and FOS-BDNF are related to a complex relationship between the mentioned three genes.

As depicted in Figures 4, 5 and 6, there is a tight relationship between CREB1 and FOS regarding expression, activation, and inhibition actions. Reciprocal inhibition action between FOS and CREB1 is seen in Figure 6, which is a contradictory finding. There are various studies about CREB1-FOS regulation relationship. Lubelski et al. reported that complete inhibition of CREB1 binding to DNA only decreased by 20% of FOS activity (Lubelski, Ponzio, & Gainer, 2012). Mutation in the GNB3 gene was accompanied by essential hypertension and obesity. GNB3 also plays a role in processes which are correlated to chronic migraine (Serafini et al., 2012). GNB3 is not involved in expression action network but appears as an indirect inhibitor of insulin in inhibition action map (Figure 6). The findings indicate that a wide range of central genes is involved in migraine, but insulin is a key agent which significantly affects migraine patients. We suggest that July, August 2019, insulin be evaluated in more details as a candidate for migraine biomarker.

Migraine is a disorder, which mostly resulted from hyperactivation of several critical genes. It seems that regulation of metabolism may be an effective treatment of migraine. Finally, more experimental data are required to validate these findings.
